# *Salmonella* and Enterohemorrhagic *Escherichia coli* Serogroups O45, O121, O145 in Wheat Flour: Effects of Long-Term Storage and Thermal Treatments

**DOI:** 10.3389/fmicb.2019.00323

**Published:** 2019-02-22

**Authors:** Fereidoun Forghani, Meghan den Bakker, Jye-Yin Liao, Alison S. Payton, Alexandra N. Futral, Francisco Diez-Gonzalez

**Affiliations:** Center for Food Safety, College of Agricultural and Environmental Sciences, University of Georgia, Griffin, GA, United States

**Keywords:** wheat flour, enterohemorrhagic *Escherichia coli*, *Salmonella*, long-term viability, thermal inactivation kinetics

## Abstract

*Salmonella* and enterohemorrhagic *Escherichia coli* (EHEC) are of serious concern in wheat flour and its related products but little is known on their survival and thermal death kinetics. This study was undertaken to determine their long-term viability and thermal inactivation kinetics in flour. Inoculation was performed using mixtures of EHEC serogroups O45, O121, O145 and *Salmonella* followed by storage at room temperature (23°C) or 35°C (for *Salmonella*). Plate counting on tryptic soy agar (TSA) and enrichment were used to assess long-term survival. For thermal studies, wheat flour samples were heated at 55, 60, 65, and 70°C and cell counts of EHEC and *Salmonella* were determined by plating. The δ-values were calculated using the Weibull model. At room temperature, EHEC serovars and *Salmonella* were quantifiable for 84 and 112 days, and were detectable for the duration of the experiment after 168 and 365 days, respectively. The δ-values were 2.0, 5.54, and 9.3 days, for EHEC O121, O45, and O145, respectively, and 9.7 days for *Salmonella*. However, the only significant difference among all values was the δ-value for *Salmonella* and serogroup O121 (*p* ≤ 0.05). At 35°C, *Salmonella* counts declined to unquantifiable levels after a week and were not detected upon enrichment after 98 days. Heat treatment of inoculated wheat flour at 55, 60, 65, and 70°C resulted in δ-value ranges of 20.0–42.9, 4.9–10.0, 2.4–3.2, and 0.2–1.6 min, respectively, for EHEC. The δ-values for *Salmonella* at those temperatures were 152.2, 40.8, 17.9, and 17.4 min, respectively. The δ-values obtained for *Salmonella* at each temperature were significantly longer than for EHEC (*p* ≤ 0.05). Weibull model was a good fit to describe the thermal death kinetics of *Salmonella* and EHEC O45, O121 and O145 in wheat flour.

**HIGHLIGHTS**

- EHEC and *Salmonella* can survive for extended periods of time in wheat flour.
- Long-term storage inactivation curves of EHEC and *Salmonella* were similar.
- EHEC was more sensitive to heat than *Salmonella.*
- Weibull model was a good fit to describe thermal death kinetics of EHEC and *Salmonella.*
- Flour storage at 35°C may be a feasible method for microbial reduction.

## Introduction

A number of studies have reported that vegetative bacterial species can survive during prolonged storage in dry foods (Eglezos, 2010; Forghani et al., 2018). The traditional process given to wheat grains to produce flour does not normally involve an antimicrobial step which results in a relatively stable microbiological load during flour production (Manthey et al., 2004).

A recent report suggests that the frequency and level of flour contamination may be much higher than previously believed (Mäde et al., 2017). The presence of pathogenic bacteria poses a serious risk to consumers because of cross contamination or consumers handling habits such as ingesting raw cookie dough (Rose et al., 2012). Recently, it has been clear that cereal foods also have the potential to transmit bacterial pathogens such as *Salmonella* and enterohemorrhagic *Escherichia coli* (EHEC).

The first account of an outbreak with *Salmonella* serovar Paratyphi, potentially linked to wheat flour was reported in Australia in 1952 (Eglezos, 2010). Since then, *Salmonella* has caused outbreaks and recalls in raw flour and its related products around the world (Centers for Disease Control and Prevention, 1998; Marler, 2006; Neil et al., 2012; McCallum et al., 2013). The FDA declared that flour and its derivatives could not be considered as ready-to-eat foods (RTE) due to the possibility of *Salmonella* presence (U.S. Food & Drug Administration, 2005). Due to the increased outbreak incidence, *Salmonella* has been considered a major pathogen in low water activity (a_w_) foods and has been extensively studied (Sperber, 2007; Centers for Disease Control and Prevention, 2009; Ma et al., 2009). However, not much is known about its survival and heat tolerance in wheat flour.

More recently, EHEC have also become pathogens of concern in wheat flour. EHEC were first recognized during the 1980s and have been predominantly linked to beef and fresh produce (Riley et al., 1983). EHEC includes multiple Shiga toxin-producing *E. coli* serogroups including the most virulent serovars O26, O45, O103, O111, O121, O145, and O157 (Mondani et al., 2014; Wang et al., 2016). The connection between beef and EHEC O157 has been well established, but other serovars have been involved in cases with other food items (Ludwig et al., 2002). However, the information about their presence in wheat flour has been rather scarce (Richter et al., 1993; Berghofer et al., 2003; Aydin et al., 2009; Sabillón Galeas, 2014).

The first flour related EHEC outbreak in the United States was due to *E. coli* O157:H7 in 2009. Tainted ready-to-bake cookie dough caused 77 illnesses, 35 hospitalizations, and 10 HUS cases (Neil et al., 2012). In 2016, EHEC O26 and O121 were associated in the first wheat flour outbreak caused by non-O157 EHEC in the United States (Crowe et al., 2017). Sixty-three patients with bloody diarrhea cases were reported in 24 states in the United States, which led to a massive flour recall (Centers for Disease Control and Prevention, 2016). The following year, two separate gastroenteritis outbreaks linked to wheat flour contaminated with EHEC O121 were reported in Canada (Canadian Food Inspection Agency, 2017a). These outbreaks resulted in several recalls and an increased concern for wheat flour safety. The most recent EHEC-related event was reported in Canada in 2017 with contaminated pie and tart shells resulting in recall (Canadian Food Inspection Agency, 2017b).

In an effort to advance our understanding about the risk of EHEC in wheat flour, we recently reported on the long-term survival and thermal inactivation kinetics of EHEC O26, O103, and O157 in wheat flour (Forghani et al., 2018). That study helped assess the likelihood of EHEC survival during storage, helping build fundamental knowledge on the subject and also tested the viability of EHEC reduction in flour by thermal treatments using linear and Weibull models (Santillana Farakos et al., 2013). It also clearly showed the superiority of Weibull model over linear model to fit thermal death kinetics of EHEC in wheat flour.

In the present study, we determined the long-term survival of EHEC O45, O121, and O145, the remaining three members of the major non-O157 EHEC group to date in wheat flour (Beier et al., 2016). Furthermore, we included *Salmonella* in the study for comparison as the best characterized pathogen related to dry foods due to its ability to persist for long times under desiccation and also its heat resistance in low a_w_ matrices (Lambertini et al., 2016). Finally, pulsed-field gel electrophoresis (PFGE) was applied to assess the frequency of strain recovery in select serogroups during long-term storage to identify survival differences among individual strains.

## Materials and Methods

### Bacteria and Growth Conditions

Fifteen EHEC and five *Salmonella* strains were used in this study including five strains of each EHEC serogroup: O45 (TW09183, TW10121, TW14003, TW07947, TW00965), O121 (TW08980, TW07927, TW08039, I2016000899, I2016012950), and O145 (GS G5578620, 4865/96, TW08087, TW09153, TW05149). EHEC strains I2016000899, and I2016012950 were obtained from the Minnesota Department of Health^1^. All other strains were obtained from the Michigan State University STEC Center^2^. Four *Salmonella* strains (*S.* Typhimurium 2009K-0300, *S.* Agona SLR141, *S. enteritidis* 2415, *S.* Anatum 6802) were provided by our culture collection at the Center for Food Safety. The last strain, *Salmonella* Typhimurium (ATCC 14028) was purchased from the American Type Culture Collection^3^.

Strains stock cultures were stored at −70°C in tryptic soy broth (TSB; Difco Laboratories, Sparks, MD, United States) supplemented with 20% (vol/vol) glycerol. All bacterial cultures were subjected to two consecutive transfers (24 h at 37°C) before use. Working stocks of each strain were prepared using Luria-Bertani broth (LB; Difco Laboratories, Sparks, MD, United States), stored at 4°C and refreshed on a monthly basis. Inoculation cultures were prepared using these stocks.

### Inoculum Preparation

Each strain was inoculated into 40 mL tryptic soy broth (TSB; Difco Laboratories, Sparks, MD, United States), and incubated for 24 h at 37°C with 200 rpm shaking, reaching stationary phase at an approximate concentration of 9 Log CFU/mL. After centrifugation (3,000 × *g*, 15 min, 4°C) cell pellets were resuspended in 10 mL sterile 0.1% buffered peptone water (BPW). Upon mixing into two 25 mL suspensions of 5-strain cocktail, suspensions were centrifuged (3,000 × *g*, 10 min, 4°C), supernatants were removed, and the remaining pellets were harvested in 200 μl 0.1% BPW. The final concentrates were used for flour inoculation.

### Inoculation Procedure

All-purpose wheat flour with an average protein content of 10% was purchased from a retail store in Griffin, GA, United States. Background microflora of flour bags was enumerated by at least three random measurements of 1 g samples. Samples were diluted in 9 mL of sterile 0.1% BPW (Neogen, Inc., East Lansing, MI, United States) in test tubes and appropriate 10-fold serial dilutions were spread plated on tryptic soy agar (TSA; Difco). After incubation (24 h at 37°C), bacterial numbers were counted and transformed to Log CFU/g. Flour samples with background flora of ≤2 Log CFU/g were used for the experiments. The pre-inoculation a_w_ of flour was measured at room temperature (23 ± 1°C) using a water activity meter (AQUA LAB Model 3TE, Decagon Devices, Pullman, WA, United States).

The concentrated cell suspensions of *Salmonella* and EHEC cocktails were aseptically spot inoculated into 15-g flour portions in a sterile stomacher bag (Nasco Whirl-Pak, Janesville, WI, United States) and hand mixed for 5 min. If necessary, mixing was continued until no clumps were observed. Subsequently, 135 g flour were added to the seeded flour samples and mixed for 3 min by hand. Finally, two sets of stomaching (Seward Stomacher, 400 Lab System, Norfolk, United Kingdom) for 3 min at 260 rpm and a 3 min manual mix were performed.

The developed inoculation procedure resulted in consistent inoculum levels of approximately 8 Log CFU/g. The a_w_ was measured after each inoculation as mentioned above. These flour samples (150 g) were used for the long-term survival studies. For thermal inactivation studies the exact same procedure was performed on 100 g of flour (10 g flour was seeded + 90 g).

### Packing and Long-Term Storage

Stomacher bags of inoculated samples were tightly closed, put inside of a Ziploc^®^Brand vacuum sealer bag and sealed without vacuuming. Sealed bags of all four sample groups (in duplicate) were put in tightly closed plastic containers and stored at room temperature (23 ± 1°C). For *Salmonella*, an additional storage at 35°C was performed to study the effect of storage temperature.

Samples were taken on days 0, 3, 7, 14, 21, and 28 followed by biweekly sampling at day 42, day 56, and then every 4 weeks. After reaching the limit of quantification (2 Log CFU/g) by standard microbiological plating, samples were enriched before detection. All bags were re-sealed immediately after each sampling. The last sampling time was 168 days (24 weeks) and 1 year (52 weeks) for EHEC and *Salmonella*, respectively. Sampling stopped after three consecutive negative results were obtained.

### Thermal Inactivation Studies

Thermal processing was performed using a digital dry bath (Fisher Scientific, Pittsburgh, PA, United States) in 0.5 mL thin wall PCR tubes with flat caps (Axygen Biosciences, Union City, CA, United States). Treatment temperatures used were 55 (up to 240 min), 60 (up to 80 min), 65 (up to 70 min) and 70°C (up to 60 min) to obtain thermal death curves for *Salmonella* and EHEC O45, O121, O145. Each particular temperature treatment was performed on 1 day, beginning from day 3, post-inoculation.

Based on our preliminary studies (data not shown) for each target temperature the dry bath block temperature was set two degrees higher, resulting in the exact desired temperature inside the tubes. Temperature inside tubes was verified at five random locations using a thermocouple bead wire temperature probe attached to a portable thermometer (HH series; OMEGA Engineering Inc. Stamford, CT, United States). Subsequently, come-up times were measured.

Inoculated flour samples (0.33 ± 0.02 g) with each five-strain *Salmonella* or EHEC cocktail were treated at once with the same time intervals for each specific temperature. Following thermal treatment the tubes were immediately placed in ice-water for one min to stop inactivation before being subjected to microbiological analyses. In addition, after the longest treatment time in each thermal treatment temperature, contents of three tubes (approximately 1 g) were rapidly transferred to the water activity meter plastic sample containers and their water activities were immediately measured.

### Microbiological Analyses

Triplicate 1 g portions were taken from duplicate 150 g flour bags at each sampling interval, 10-fold serial dilutions in 0.1% BPW (Neogen, Inc., East Lansing, MI, United States) were prepared and spread plated on TSA. Following incubation at 37°C for 24 h, colonies were counted and transformed into Log CFU/g for microbiological analysis.

Un-inoculated flour samples were tested to determine background aerobic plate count. After reaching the limit of detection without enrichment, three 1-g samples from each stored bag were transferred to 40 mL of lauryl tryptose broth (LTB; Difco) or TSB (Difco) for EHEC and *Salmonella*, respectively, and enriched for 24 to 48 h at 37°C with 200 rpm shaking.

Appropriate dilutions of the pre-enriched EHEC homogenates were spread plated on sorbitol MacConkey agar (SMAC; Difco) supplemented with 0.05 mg/L cefixime and 2.50 mg/L potassium tellurite (Sigma-Aldrich, Inc., St. Louis, MO, United States) (CT-SMAC) (Ludwig et al., 2002) or loaded onto 3M Petrifilm *E.* coli/Coliform Count Plate (6404, 3M Microbiology, St. Paul, MN, United States) according to the manufacturer’s guidelines and incubated at 37°C for 24 h. For *Salmonella*, xylose lysine deoxycholate agar (XLD; Difco) was used.

All presumptive EHEC colonies recovered from TSA and CT-SMAC were confirmed using serogroup specific agglutination test (Cedarlane, Burlington, ON, Canada). For *Salmonella* black colonies on XLD agar were further confirmed as *Salmonella*, using agglutination test as well (*Salmonella* H Antiserum Poly a-z; Difco). For the analysis of thermally treated samples, tube contents were transferred to 9.7 mL 0.1% BPW and appropriate 10-fold serial dilutions were spread plated on TSA for microbial enumeration. Colony counts were transformed to Log CFU/g and used for data analysis.

### Data and Statistical Analysis

Survival counts were calculated according to Food and Drug Administration’s Bacteriological Analytical Manual’s formula for aerobic plate counts modified for 0.1 mL plating volumes (Maturin and Peeler, 1998). Average values of triplicate measurements at each sampling time were used with a minimum of two replicate trials for each test (*n* = 6).

Weibull model (Mafart et al., 2002) was fit to the obtained data using Microsoft Excel 2016 Add-in GinaFit Version 1.7 (Geeraerd et al., 2005).

Weibull model (Mafart et al., 2002):

$$\log(N_{\text{t}})=\log(N_{0})-{\left(t/\text{δ}\right)}^{\text{β}}$$

Based on the definitions by Mafart et al. (2002), *N*_t_ is the population at time *t* (CFU/g), *N*_0_ is the population at time 0 (CFU/g), δ is the time required for the first decimal reduction (min) and β is a fitting parameter that describes the shape of the curve (β > 1 convex, β < 1 concave). Finally, to further evaluate the developed Weibull model, the equation was solved using *N*_0_ obtained from the actual data and δ- and β-values obtained from the model for each data point.

While decimal reduction means a 90% reduction in the survival counts, parameter δ is distinguished from the conventional *D*-value since it shows the first decimal reduction while in contrast *D*-value which is derived from the linear first-order kinetic represents the time of decimal reduction, regardless of the heating time.

Water activities of all flour bags used, means of inactivation parameters in survival counts (Log CFU/g) from long-term storage and thermal inactivation tests were subjected to *t*-test and analysis of variance (ANOVA), respectively. Tukey’s multiple range test was applied for the long-term survival and analysis of values obtained from the same temperature treatments. Fisher’s least significant difference (LSD) test was applied for the comparison of different treatment temperatures in each serogroup. All analyses were performed using IBM SPSS Statistics Version 24 (SPSS Institute, Chicago, IL, United States). The significance of difference was defined at *p* ≤ 0.05.

### Pulsed-Field Gel Electrophoresis

Pulsed-field gel electrophoresis was performed using the standardized PulseNet’s laboratory protocol^4^ with minor modifications on two select serogroups. Random single colonies of EHEC O121 at days 21, 42, 56, and 84 of storage and EHEC O45 at day 168 of storage were selected for PFGE. Subsequently, PFGE was performed on these colonies along with the five representative strains of each individual serogroup for identification using comparison of PFGE band patterns.

Briefly, single colonies grown in TSB (Difco) at 37°C overnight were streaked onto TSA with 5% sheep blood (Northeast Laboratory, Waterville, ME, United States) and incubated at 37°C for 14–18 h. Using sterile swabs, bacterial colonies were suspended in 2 mL of cell suspension buffer (100 mM Tris, 100 mM EDTA, pH 8.0) in 12 mm × 75 mm tubes (Falcon 2054; Corning, NY, United States). Cell concentrations were adjusted to optical density of 0.45 at 610 nm wavelength using a spectrophotometer (MicroScan Turbidity Meter, Simens Healthcare Diagnostics Inc., West Sacramento, CA, United States).

Following concentration adjustment, 400 μL cell suspensions were mixed with 400 μL of melted 1% SeaKem Gold Agarose (SKG; Lonza, Basel, Switzerland) cooled to 60°C and 20 μL of 20 mg/mL Proteinase K (Amresco LLC., Solon, OH, United States) by brief gentle pipetting in 1.5 mL microcentrifuge tubes. The mixtures were immediately dispensed into wells of reusable plug molds (Bio-Rad Laboratories, Hercules, CA, United States) and stored for 15 min at room temperature to solidify. Solidified plugs were trimmed and carefully transferred to 50 mL conical tubes containing 5 mL of cell lysis buffer (50 mM Tris, 50 mM EDTA, pH 8.0, 1% sarcosyl) with 0.1 mg/mL Proteinase K (Amresco; 25 μL of 20 mg/mL stock solution) and incubated at 55°C with constant vigorous agitation (200 rpm).

After lysis, plugs were washed twice using 15 mL of sterile ultrapure water pre-heated to 55°C with shaking (200 rpm) for 15 min and subsequently with 15 mL of TE buffer (10 mM Tris, 1 mM EDTA, pH 8.0) for four times using the same conditions. After the last TE wash, plugs were stored in 5 mL TE buffer at 4°C until restriction digestion. Restriction digestion was performed using *Xba*I restriction enzyme (Roche Diagnostics, Indianapolis, IN, United States).

**Table 1:** Rate of enterohemorrhagic *E. coli* and *Salmonella* inactivation in wheat flour stored at room temperature (23 ± 1°C) for 84 days (12 weeks) and 112 days (16 weeks), respectively, calculated by the Weibull model ^1^.

	Weibull model parameters				
8 Bacteria	δ(days)	δSE	β	βSE	Adj.*R*^2^
EHEC O45	5.54^ab^	1.17	0.57	0.04	0.97
EHEC O121	2.04^a^	0.08	0.46	0.00	0.93
EHEC O145	9.34^ab^	5.2	0.70	0.16	0.98
*Salmonella*	9.67^b^	3.06	0.56	0.07	0.91

*^1^Within each column, values followed by the different lowercase letters are significantly different (p ≤ 0.05).*

The plugs were cut into 2.5 mm slices and transferred to 1.5 mL microcentrifuge tubes for restriction digestion. A pre-restriction incubation step was performed in which each plug slice was placed in 200 μL of 1× restriction buffer and incubated at room temperature for 15 min. Subsequently, pre-restriction buffer was removed and restriction digestion was performed in 200 μL of digestion master mix consisted of 173 μL ultrapure water, 20 μL 10× restriction buffer, 2 μL of 10 mg/mL bovine serum albumin and 5 μL of restriction enzyme (50 U total) incubated at 37°C for 2 h.

The enzyme/buffer mixtures were removed and after 5 min incubation in 0.5× Tris Borate EDTA (TBE) at room temperature fixed at the bottom of comb teeth for 15 min followed by gel casting. The 1% SKG agarose (55°C) was poured and left to solidify for 45 min. After solidification, DNA restriction fragments were separated with a CHEF-DR^®^III (Bio-Rad) with pulse times with settings as 6.76 s initial switch time, 35.38 s final switch time, 6 V/cm voltage, 120° included angle or CHEF Mapper (Bio-Rad) using the auto algorithm for non-O157 Shiga toxin-producing *E. coli*. All PFGE runs were performed at 14°C for 18 h and 20 min with a 0.5× TBE flow rate of 1 L/min. *Salmonella* ser. Braenderup H9812 was used as standard control in all experiments.

Gels were stained using SYBR^®^Safe (S-33102, Invitrogen, Carlsbad, CA, United States) and restriction fragment patterns were photographed by a Gel Doc^TM^ XR^+^ imaging system (Bio-Rad) using an Image Lab^TM^ Software. The final images were analyzed with BioNumerics software version 6.6 (Applied Maths Inc., Sint-Martens-Latem, Belgium) and fingerprints data were clustered for identification of randomly selected colonies after long-term storage.

## Results

### Background Microbiota and a_w_ of Wheat Flour

The background aerobic plate count of all flours used in the study was confirmed as 2 Log CFU/g or less. All flour samples used for long-term survival had a_w_ of 0.50 and 0.57 pre- and post-inoculation, respectively. This was equivalent to maximum a_w_ differences of approximately 0.09 between pre- and post-inoculation. In flour samples used for thermal inactivation tests a_w_ values measured pre- and post-inoculation were 0.45 and 0.52, respectively, showing an a_w_ difference of less than 0.09. No significant difference was observed between the a_w_ of the flour samples bought or between samples pre- and post-inoculation. Samples a_w_ did not change during storage (data not shown).

The standard deviation of the counts obtained from six post-inoculation subsamples (1 g each) of each EHEC serogroup and *Salmonella* in each measurement was ≤0.3 Log CFU/g, confirming the homogeneous inoculum mixing during inoculation process. Also, measurements before and after the thermal treatments at their longest time point resulted in less than 0.052 change in a_w_, regardless of treatment temperature.

### Long-Term Survival of *Salmonella* and EHEC in Flour

**FIGURE 1:**
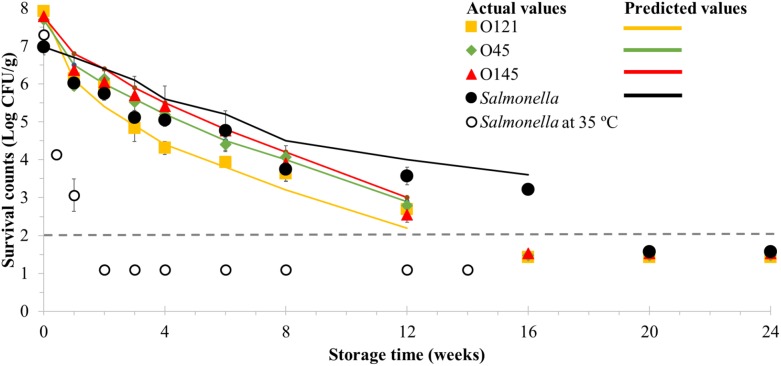
Long-term survival of enterohemorrhagic *Escherichia coli* serogroups O45, O121, O145 and *Salmonella* in wheat flour at room temperature (23 ± 1°C) for 24 weeks and *Salmonella* at 35°C (14 weeks). Time points shown below the detection limit (dashed line) by direct plating (2 Log CFU/g) indicate positive samples after enrichment. Room temperature samples were enriched from weeks 16 and 20 of storage for EHEC and *Salmonella*, respectively. Values are the Log-transformed number of surviving cells per gram of sample, shown as the mean of at least three independent trials with minimum two replicates with standard error of the mean indicated. Data markers represent the actual values and lines represent the predicted surviving cell numbers calculated by solving the Weibull equation, using δ- and β-values obtained from the model for each data point and the actual *N*_0_ based on actual survival counts (data markers).

There were approximately 1 Log CFU/g reductions of the initial viable counts after the first 4 days followed by approximately 0.5 Log CFU/g declines up to 10 days of storage for the three EHEC serogroups and *Salmonella* stored at room temperature (Figure 1). During the subsequent 4 weeks the reduction rates slowed down to approximately 0.5 Log CFU/g per week, and then to approximately 0.3 Log CFU/g per week until day 84 of storage, which was the last time point that surviving EHEC was quantified by direct plating.

Between weeks 8 and 16, the *Salmonella* survival rate had a slower decline compared to EHEC, which resulted in its quantification at least 4 weeks beyond. Viable cells of EHEC O45, O121, and O145 and *Salmonella* were still detected in wheat flour at 168 days of storage at room temperature (23 ± 1°C) which was the last time point of sampling for EHEC (Figure 1). In case of *Salmonella*, positive samples were still recovered after 1 year, which was the last sampling time point for *Salmonella*. The fastest reductions in both viable EHEC and *Salmonella* were observed in the early post-inoculation days. All EHEC serogroups had similar survival curves with multiple overlapping time points within the time-course curves. Furthermore, the same trend was also observed for *Salmonella* up to week 8 of storage.

Wheat flour inoculated with *Salmonella* was also stored at 35°C and the viability was determined (Figure 1). A decline of more than approximately 1 Log CFU/g was observed in the first 4 days of storage and samples after 1 week of storage were the last ones to be quantifiable without enrichment. Enrichment was performed from the second week of storage and resulted in positive recovery of *Salmonella* during no more than 98 days (14 weeks) of storage.

### Long-Term Inactivation Kinetics of EHEC and *Salmonella* in Wheat Flour

The calculation of the parameters of inactivation kinetics of EHEC and *Salmonella* stored at room temperature (23 ± 1°C) were determined with data points obtained during 84 and 112 days, respectively (Table 1). First decimal reduction times (δ) of serogroups O45, O121, O145 and *Salmonella* as 5.54, 2.04, 9.34, and 9.67 days, respectively, were calculated using the Weibull model.

While none of the δ-values were significantly different (*p* > 0.05) among the EHEC serogroups, the *Salmonella* δ-value was significantly different from one of the serogroups, O121 (*p* ≤ 0.05). The Adj. *R*^2^ values of the Weibull model were in the range of 0.91–0.97, clearly showing the good fit of Weibull model for EHEC and *Salmonella* death kinetics during long-term storage at room temperature (23 ± 1°C).

### Thermal Inactivation Kinetics of EHEC and *Salmonella* in Wheat Flour

In general, come-up times were shorter as temperatures increased gradually. Briefly, at 60, 65, and 70°C it took samples between 30 and 50 s to reach to 1–2°C lower than the desired temperature. From there, a maximum of 30 s time was needed to reach the unified set temperature throughout the sample inside the 0.5 mL tubes (60, 65, 70°C). The come-up time was a little longer for the 55°C samples compared to the other higher temperatures. It took approximately 90 s to reach 1–2°C lower than the desired temperature and another 30 s to reach the exact temperature (55°C).

**FIGURE 2:**
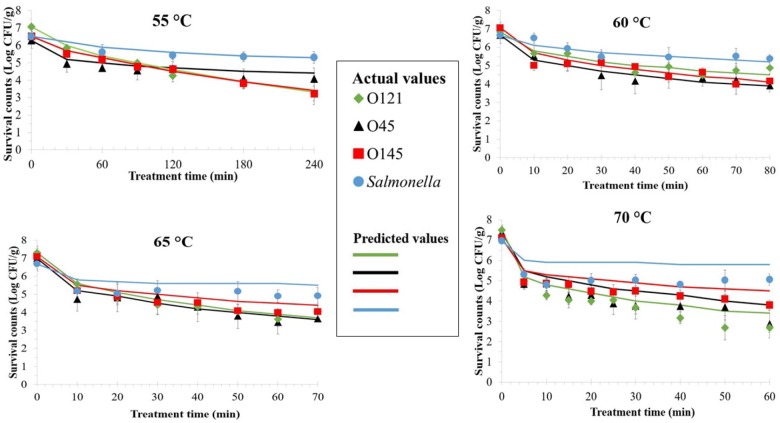
Thermal inactivation curves of enterohemorrhagic *Escherichia coli* serogroups O45, O121, O145, and *Salmonella* in wheat flour at 55, 60, 65, and 70°C. Values are the Log-transformed number of surviving cells per gram of sample, shown as the mean of at least three independent trials with minimum two replicates with standard error of the mean indicated. Data markers represent the actual values and lines represent the predicted surviving cell numbers calculated by solving the Weibull equation, using δ- and β-values obtained from the model for each data point and the actual *N*_0_ based on actual survival counts (data markers).

The viable counts of all serovars and *Salmonella* declined more rapidly after inoculated flour samples were heated at any temperature at the beginning, but the reduction rate slowed down later during incubation (Figure 2). This trend was more noticeable for *Salmonella*, when its counts remained almost similar throughout the treatment period after the initial decrease. The three EHEC serovars had inactivation curves that resembled each other at each particular temperature. Overall, after 60 min of thermal treatment at 55, 60, 65, and 70°C average microbial count reductions of 1.57, 2.36, 3.44, and 4.22 Log CFU/g for the three EHEC combined, and 0.90, 1.14, 1.79, and 1.91 Log CFU/g for *Salmonella*, were achieved, respectively.

The Weibull model was employed to determine the thermal inactivation kinetics parameters of EHEC serogroups and *Salmonella* at 55, 60, 65, and 70°C (Table 2). Same as in room temperature storage Weibull model was a good fit. The Adj. *R*^2^ values for EHEC δ-values were in the range of 0.85–0.98. The same values were in the range of 0.71–0.94 for *Salmonella*.

No significant difference was observed among the δ-values of different EHEC serogroups at each treatment temperature obtained by Weibull model. However, for *Salmonella* δ-values were always significantly different from EHEC (*p* ≤ 0.05), regardless of the treatment temperature. The 5°C increments in treatment temperature both for each EHEC serogroup as well as *Salmonella* always resulted in decline of δ-values, but the differences at higher temperatures were not always significant. At 70°C, the δ-values were 1.55, 0.17, 0.76, and 17.36 min for serogroups O45, O121, O145, and *Salmonella*, respectively.

**Table 2:** Thermal inactivation rates of enterohemorrhagic *E. coli* and *Salmonella* in wheat flour calculated by Weibull model.

Bacteria		Weibull model parameters				
	Temp (°C)	δ (min)	δ SE	β	β SE	Adj. *R*^2^
EHEC O45	55	^B^20.04^a^	8.06	0.26	0.09	0.87
	60	^AB^5.24 ^a^	2.35	0.37	0.06	0.87
	65	^A^2.76*^a^*	0.86	0.38	0.03	0.85
	70	^A^1.55**^a^**	1.51	0.31	0.13	0.92

EHEC O121	55	^B^26.57^a^	9.14	0.60	0.10	0.98
	60	^AB^10.06^a^	2.17	0.41	0.00	0.93
	65	^A^3.19*^a^*	1.29	0.42	0.07	0.95
	70	^A^0.17**^a^**	0.11	0.24	0.03	0.95

EHEC O145	55	^B^42.86^a^	2.41	0.66	0.01	0.98
	60	^A^4.86^a^	0.82	0.38	0.02	0.89
	65	^A^2.37*^a^*	0.11	0.29	0.03	0.95
	70	^A^0.76**^a^**	0.75	0.20	0.09	0.93

*Salmonella*	55	^A^152.22^b^	125.32	0.27	0.07	0.94
	60	^A^40.78^b^	14.63	0.46	0.09	0.82
	65	^A^17.89*^b^*	16.14	0.11	0.05	0.83
	70	^A^17.36**^b^**	17.00	0.04	0.04	0.71

*Values marked with different uppercase letters within each individual EHEC serogroup and Salmonella are significantly different. Values for the same treatment temperature (55°C: ^a^, 60°C: ^a^, 65°C: a, 70°C: **a**) within each column followed by different lowercase letters are significantly different (p ≤ 0.05).*

### PFGE Pattern Analysis

**FIGURE 3:**
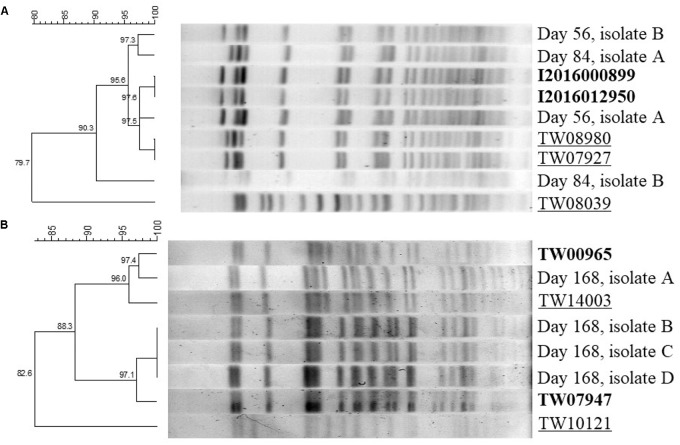
Dendrogram of two EHEC isolates of serogroup O121 at days 56 and 84 of storage **(A)** and serogroup O45 at day 168 of storage **(B)** on pulsed-field gel electrophoresis (PFGE). Bold types represent the previously known patterns of the isolates with maximum similarity to post-storage random isolates. Light types represent the single isolates randomly selected after long-term storage. Underlined types represent the remaining standards that were not recovered at selected random isolates upon storage.

The PFGE dendrogram of randomly selected EHEC isolates from serogroup O121 at days 56 and 84 of storage and serogroup O45 at day 168 of storage along with the patterns obtained from the known isolates of each serogroup were used for identification (Figure 3). For serogroup O121, the two isolates from day 56 and the two from day 84 of storage were all identified as either I2016000899 or I2016012950 (Figure 3A). In case of EHEC serogroup O45, one of the random isolates from day 168 of storage was identified as TW00965 and all the other three as TW07947 (Figure 3B).

## Discussion

The information available about EHEC long-term survival in wheat flour was very limited until our recent study (Forghani et al., 2018), in which we reported survival and thermal inactivation of EHEC serovars O26, O103, O111, and O157 in wheat flour. Such information was important to the industry due to the fact that product composition such as the unique composition of wheat flour may affect the rate of microbial inactivation (Santillana Farakos et al., 2014). The EHEC (O26, O103, O111, and O157) survived in wheat flour stored at room temperature (23 ± 1°C) for 1 year and survival was much lower as storage temperature increased to 35°C. This finding was in agreement with the previously reported results that indicated the survival of bacteria was favored by reduced a_w_ and temperature (Beuchat et al., 2013).

To further expand the knowledge on EHEC risk assessment of wheat flour and to be able to better predict their process lethality, in the present study three additional EHEC serogroups not included in the previous study (O45, O121, O145) were assessed. Also, *Salmonella* was included for comparison as a major pathogen of concern in dry foods including flour and related products. Thus in the present paper, we included assessing *Salmonella* long-term survival at room temperature and thermal death kinetics at 55, 60, 65, and 70°C in wheat flour. In addition, *Salmonella* survival was studied at 35°C for comparison with similar data on EHEC obtained in our previous study (Forghani et al., 2018). These temperatures were selected because it was unlikely that they had negative influence on the wheat flour properties (Ozawa et al., 2009; Khamis, 2014; Sudha et al., 2016) for the amount of time that they were used in the study.

Due to the importance of inoculation method in challenge food studies and in particular dry foods (Hildebrandt et al., 2016; Esbelin et al., 2018; Limcharoenchat et al., 2018) a method based on application of concentrated cell suspension for inoculation was developed in our previous study (Forghani et al., 2018). This method could efficiently deliver as high as approximately 8 Log CFU/g inoculum level homogeneously, did not show variabilities which are of concern for inoculation (Blessington et al., 2013; Beuchat et al., 2017) and barely changed the water activity of post-inoculation wheat flour. We also considered that it was important to maintain the same conditions for EHEC and *Salmonella* to maximize the accuracy of comparisons.

Reduction of a_w_ is one of the main strategies to control microbial contamination and growth in food matrices due to inhibition of enzymatic reactions and metabolism in low a_w_ (Maserati et al., 2017). It is also well established that a_w_ is a key factor influencing the survival of *Salmonella* in thermal processing as increase in a_w_ will result in decrease in the thermal resistance (Xu et al., 2018). Furthermore, decreasing a_w_ may also enable bacteria to survive longer (Podolak et al., 2012).

*Salmonella* long-term survival was previously reported in other low a_w_ matrices such as peanut flour (Kataoka et al., 2014), pasta (Rayman et al., 1979), raw pecan and peanut kernels (Brar et al., 2015) stored at room temperature for 1 year. It was also reported to survive in cracker sandwiches and cookie stored at 25°C for at least 182 days (Beuchat and Mann, 2015). The present work advanced our understanding on *Salmonella* long-term survival in wheat flour reporting that flour was still positive for *Salmonella* after 1 year post-inoculation at 23°C.

*E. coli* O157, O111 and O26 were previously reported to survive for 113 days in mallow stored at 22°C at a a_w_ of 0.73 with inoculum levels of 4 Log CFU/g. In the same study, survival was only 90 and 42 days for chocolate (a_w_: 0.40) and biscuit cream (a_w_: 0.75), respectively, emphasizing the effect of matrix type on survival (Baylis et al., 2004). In other studies *E. coli* O157:H7 survived on walnut kernels at 23°C (Blessington et al., 2012) and peanut and pecan kernels at 22°C for at least 12 months (Brar et al., 2015). Results from our previous study (Forghani et al., 2018), in agreement with the above mentioned reports revealed that EHEC O26, O103, O111, and O157 survived for at least 1 year in wheat flour stored at 23°C. In the present study, the inactivation curves of EHEC O45, O121, and O145 perfectly aligned with our previous study and therefore sampling was stopped at day 168 of storage.

The long-term survival of pathogens in flour should be of further concern to the food safety specialists and industry as *Salmonella* and *E. coli* may not be the only sources of risk. For example, a recent study reported extended stability of *Listeria monocytogenes* in wheat flour for up to 210 days at room temperature, as well as its enhanced thermal resistance in flour with 0.31 and 0.56 a_w_ (Taylor et al., 2018). Long-term inactivation kinetics of EHEC O45, O121, and O145 were consistent among serogroups. Based on the adjusted *R*^2^ values obtained, in agreement with previous studies, in this study the Weibull model was found to be suitable for studying the death kinetics of EHEC and *Salmonella* in wheat flour (Ma et al., 2009; Santillana Farakos et al., 2014; Rachon et al., 2016; Forghani et al., 2018).

Generally, survival of bacteria in low a_w_ foods is reduced as temperature increases (Beuchat et al., 2017). For instance, *E. coli* survived in chocolate (a_w_: 0.40) for 366, 90 and 43 days at 10, 22, and 38°C, respectively (Baylis et al., 2004). In wheat flour, while samples were still positive for EHEC after a year of storage at 23°C, the last positive samples at 35°C for EHEC O26 and O157 upon enrichment were observed at 28 and 49 days, respectively. Interestingly, *Salmonella* storage at 35°C resulted in reductions comparable to the ones from EHEC in our previous study (Forghani et al., 2018), especially in terms of both EHEC and *Salmonella* reaching unquantifiable range after only a week, post-inoculation. The only difference was that *Salmonella* was positive upon enrichment up to 98 days which was twice the values observed for EHEC O157 but still much shorter than the 23°C storage.

These results are important due to the consistency between *Salmonella* and EHEC making flour storage at 35°C for an appropriate length of time before distribution a possible strategy for the milling industry to reduce the microbial risk for consumers. This can be a helpful strategy since thermal treatment might be harder to perform on industrial scale and it also may not be as effective on *Salmonella* as it is for EHEC.

The thermal inactivation times were calculated from the time when samples were placed in the heat block due to the minimal ratio of come-up times to our thermal treatment times and for better consistency. The δ-values for *Salmonella* at 55, 60, 65, and 70°C were 152.22, 40.78, 17.89, and 17.36 min, respectively. Similar values were reported as 9.97, 5.51, and 2.11 min at 75, 80, and 85°C, respectively, for *Salmonella* enteritidis PT 30 in wheat flour (Smith et al., 2016). The same study also reported δ-values of 8.08, 4.97, and 1.59 min at 75, 80, and 85°C, respectively. Interestingly, it was recently reported that in a closed system, the a_w_ of flour will significantly increase during the thermal treatment. That study reported that flour with an a_w_ of 0.45 at 25°C reached an a_w_ of approximately 0.71 at 80°C, in a closed system (Liu et al., 2018).

In another study δ-value of 5.1 min for pre-fabrication inoculated flour and 4.7 min, for post-fabrication inoculated flour both at 80°C were reported, respectively, emphasizing the effect of inoculation protocol on *Salmonella* thermal death kinetics (Limcharoenchat et al., 2018). However, defining the inoculation protocol that is the closest simulation of real contamination routes based on product type and pathogen of concern remains a challenging subject for further discussion and future research.

The thermal death kinetics of EHEC serogroups obtained in this study (O45, O121, O145) were similar to those obtained in our previous study for EHEC O26, O103, O111, and O157, confirming those observations. The data presented in these two works can be a useful foundation consisting of seven different serogroups information to predict EHEC behavior upon thermal treatment at 55, 60, 65, and 70°C in wheat flour. Hiramatsu et al. (2005) also reported that *E. coli* dried on paper disks (a_w_: 0.56) partially survived after 5 h exposure to 70 and 80°C.

As expected, the Weibull model was a good fit overall to describe the thermal death kinetics of EHEC and *Salmonella* in flour in addition to their long-term survival which has also been previously reported in dry foods (Ma et al., 2009; Santillana Farakos et al., 2014; Forghani et al., 2018). The suitability of Weibull model was further confirmed by solving the Weibull equation, using δ- and β-values obtained from the model for each data point and the actual *N*_0_, resulting in close predictions as shown in Figure 1, 2.

Higher variabilities were observed for *Salmonella* in comparison to EHEC resulting in lower Adj. *R*^2^ and greater SE values in general. Such variations have also previously been reported for *Salmonella* by Blessington et al. (2013); Beuchat et al. (2017), and Limcharoenchat et al. (2018), who attributed the variations to inoculation method. In an extended study focusing on the effects of inoculation procedures on *Salmonella* thermal resistance in wheat flour, Hildebrandt et al. (2016) reported that inoculation method impacted repeatability, population stability and inactivation kinetics of *Salmonella* in wheat flour, regardless of laboratory. It would be a good subject for future studies to assess the possible reasons for such variations.

Another interesting topic for future works would be to identify and study the mechanisms involved in enhanced resistance of *Salmonella* and EHEC upon dehydration. Although the effect is well known and has been reported in many studies, the information available on the mechanisms involved remains rather scarce. It has been suggested that the complex resistance response during dehydration may involve a coordinated set of mechanisms such as removal of oxygen species, accumulation of amphiphilic solutes and/or proteins, down-regulation of metabolism and accumulation of disaccharides, such as trehalose and sucrose (França et al., 2007). The latter is known as the water replacement hypothesis and has been mentioned as a possible mechanism in *Salmonella* and Shiga-toxin producing *E. coli* (Hiramatsu et al., 2005).

Deng et al. (2012) performed a transcriptome sequencing of *Salmonella* under desiccation and starvation stress concluding that under desiccation *Salmonella* cells were in dormant state with only <5% of the genome being transcribed compared to 78% in LB broth. In their study, they reported that genes involved in heat and cold shock response, DNA protection and regulatory functions likely play roles in cross protecting *Salmonella* from desiccation stresses.

Production of osmoprotectants, alternative sigma factors, rRNA degradation, viable but non-culturable state (VBNC) and filamentation are some other strategies that have been suggested to be used by *Salmonella* to adapt to desiccation (Finn et al., 2013). More recently, Maserati et al. (2018) reported a proteomic analysis of *Salmonella* in response to desiccation, low a_w_ and thermal treatment indicating that ribosomal proteins might be important for additional regulation of the cellular response under these conditions. In another work, they also reported that virulence factors *sopD* and *sseD* were critical for *Salmonella* survival during desiccation (Maserati et al., 2017).

Upon analysis of the PFGE patterns it was observed that during long-term storage the ratio between the five strains used in each inoculum did not remain the same and certain strains were recovered more frequently. Interestingly, in serogroup O121 the two strains that were recovered after PFGE of randomly selected colonies at days 56 and 84 of storage (I2016000899, I2016012950) were the two related to a recent flour outbreak (Centers for Disease Control and Prevention, 2016). Same strains were the ones that became dominant through long-term storage. Thus, assessment of the genomic differences between these strains and the other ones in the same serogroup as well as between serogroups would be a good target for future studies, in order to understand the risk factors enabling a strain or serogroup to better survive in flour. In agreement to serogroup O121, the same trend was observed in serogroup O45 resulting in dominance of two strains out of the initial five after 168 days of storage.

## Conclusion

In conclusion, findings of the present work further confirmed that EHEC and *Salmonella* could survive for extended periods in wheat flour stored at typical conditions used at home and commercial settings. Heat treatment is an effective method for mitigating risk of EHEC in flour but is more limited in case of *Salmonella*. On the other hand, storing produced flour at slightly high temperatures (35°C) for a minimum period of 2 months before distribution can be an effective substitute strategy affecting both EHEC and *Salmonella*. Results from this study further improve our knowledge regarding risk assessment and management, such as predicting thermal process lethality of EHEC and *Salmonella* in flour.

## Author Contributions

FF and FD-G designed the study, conceived the experiment design, analyzed and interpreted the data, and drafted the manuscript. FF, MB, J-YL, AP, and AF performed the experiments. AP also helped with editing the manuscript.

## Conflict of Interest Statement

The authors declare that the research was conducted in the absence of any commercial or financial relationships that could be construed as a potential conflict of interest.

## Footnotes

1 http://www.health.state.mn.us/

2 http://shigatox.net/stec/cgi-bin/index

3 https://www.atcc.org/

4 https://www.cdc.gov/pulsenet/pdf/ecoli-shigella-salmonella-pfge-protocol-508c.pdf
